# Reduced rank regression-derived dietary patterns related to climate-sensitive micronutrients and their associations with child undernutrition among young children in rural Kenya: findings from the ALIMUS study

**DOI:** 10.1186/s12889-026-26265-z

**Published:** 2026-01-13

**Authors:** Grace Wothaya Kihagi, Adi Lukas Kurniawan, Erick Agure, Erick M.O. Muok, Raissa Sorgho, Ina Danquah

**Affiliations:** 1 Heidelberg Institute of Global Health (HIGH), Medical Faculty and University Hospital, Heidelberg University, Heidelberg, Germany; 2 Transdisciplinary Research Area (TRA) “Technology and Innovation for Sustainable Futures” and Center for Development Research (ZEF), Rheinische Friedrich-Wilhelms University of Bonn, Beringstrasse 1, Bonn, 53115 Germany; 3 Kenya Medical Research Institute (KEMRI), Center for Global Health Research (CGHR), Kisumu, Kenya; 4 Public Health Institute (PHI), Center for Wellness and Nutrition (CWN), Sacramento, CA USA

**Keywords:** Climate-sensitive micronutrients, Dietary pattern, Children, Undernutrition, Reduced rank regression

## Abstract

**Background:**

Undernutrition among children remains a global public health challenge in sub-Saharan Africa. This study examined the associations of dietary patterns related to climate-sensitive micronutrients with undernutrition among children aged 6–23 months in Siaya County, Kenya.

**Methods:**

We used cross-sectional baseline data of 626 mother-child pairs from a cluster-randomized controlled trial on nutrition counselling and home gardening. Dietary patterns and food intake were derived from a semi-quantitative food frequency questionnaire and identified using Reduced-Rank Regression (RRR) with the response variables of iron, zinc, selenium, and vitamin A (climate-sensitive micronutrients). Their associations with anthropometric z-scores [weight-for-age (WAZ), weight-for-height (WHZ), height-for-age (HAZ)] were calculated by regression models.

**Results:**

In this study population (median age: 15 months; 54.2% boys), boys had a lower median of WAZ (-0.47 vs. -0.20), WHZ (-0.02 vs. 0.18), and HAZ (-0.88 vs. -0.54) than girls (*p* < 0.05). RRR-derived dietary patterns were similar between boys and girls, explaining 68% and 65% of the variations in micronutrient intakes, respectively. These patterns, characterized by high consumption of vegetables, fish, potatoes, coffee and tea, white bread and cereals, fruits, rice and pasta, fermented food, and legumes, were positively associated with WAZ and WHZ but not with HAZ, only among girls.

**Conclusion:**

A diet rich in protein sources and fruits and vegetables is associated with better general and acute nutritional status among young girls in rural Kenya.

**Supplementary Information:**

The online version contains supplementary material available at 10.1186/s12889-026-26265-z.

## Introduction

Undernutrition, particularly among young children, remains a major public health challenge in low- and middle-income countries, including sub-Saharan Africa [1]. Children aged 6–23 months constitute a vulnerable group, as they are in a critical period of rapid growth and development, and they rely on nutrient-dense complementary foods to meet their nutritional needs [2]. In Kenya, 22% of children under five had chronic undernutrition or stunting [3], 11% have general undernutrition or underweight, and 4% have acute undernutrition or wasting [4].

Climate change is increasingly recognized as a significant determinant of food security and undernutrition [5]. Rising global temperatures, rainfall pattern variability, and more frequent extreme weather events—such as droughts and floods—are disrupting agricultural production and crop yields, as well as threatening food systems in sub-Saharan Africa, including Kenya [6, 7]. Changes in agricultural yields due to climate change can lead to decreased availability and accessibility of nutrient-rich foods [8]. Climate-sensitive micronutrients, such as iron, zinc, selenium, and vitamin A, are essential for child growth and immune function yet are particularly at risk, because their availability can fluctuate due to climate-induced changes. Changes in food production and supply make children highly susceptible to climate-related deficiencies in these micronutrients [9]. The estimated losses of the essential nutrients in major food crops due to increased atmospheric greenhouse gases are 3 to 10% for zinc and iron [10], 9% for selenium [11], and 15% for vitamin A [12].

In Kenya, the impacts of climate change on food systems are already evident. Over the past four decades, average temperatures have increased by approximately 1 °C, with projections indicating further rises of 1.5–3 °C by mid-century [13]. In this region, where the agricultural sector forms the backbone of livelihoods and a significant portion of the population relies on small-scale rain-fed agriculture, climate-induced disruptions in food production can result in reduced dietary diversity, increased reliance on energy-dense but nutrient-poor foods, and exacerbate the risk of undernutrition among vulnerable populations [13, 14]. Understanding how dietary patterns influence the status of climate-sensitive micronutrients and their impact on undernutrition is critical, particularly in regions where food insecurity and climate change are closely linked.

Reduced rank regression (RRR) offers a promising approach to address this question by identifying dietary patterns that are strongly associated with specific intermediate markers of health. By focusing on climate-sensitive micronutrients as these response variables, RRR can identify a linear combination of predictor variables (food groups) that explains the maximum variation in pre-specified response variables, rather than merely describing food consumption patterns, thereby providing insights into how diet influences health risks [15]. Moreover, to date, limited research has explored the associations between dietary patterns that explain variations in climate-sensitive micronutrients and undernutrition among young children in rural Kenya. Therefore, the present study aimed to derive dietary patterns that explain the variations in the intakes of climate-sensitive micronutrients (iron, zinc, selenium, and vitamin A) among boys and girls, and to determine their associations with nutritional status among children aged 6–23 months in rural Kenya.

## Methods

### Study design and recruitment

This cross-sectional study used a baseline dataset from the ALIMUS – We are feeding! study, which is a cluster-randomized controlled trial [16]. Data of mother-child pairs in Siaya County, Kenya, were collected from July to December 2021. In Siaya County, the population relies mainly on subsistence farming and fishing. The primary food crops produced include maize, beans, sweet potatoes, sorghum, rice, and cassava. We adhered to the principles of the Declaration of Helsinki and obtained ethical approval from the Kenya Medical Research Institute (KEMRI) and the Medical Faculty at the University of Heidelberg, Germany. All caregivers gave informed written consent before the data collection.

**Fig. 1:**
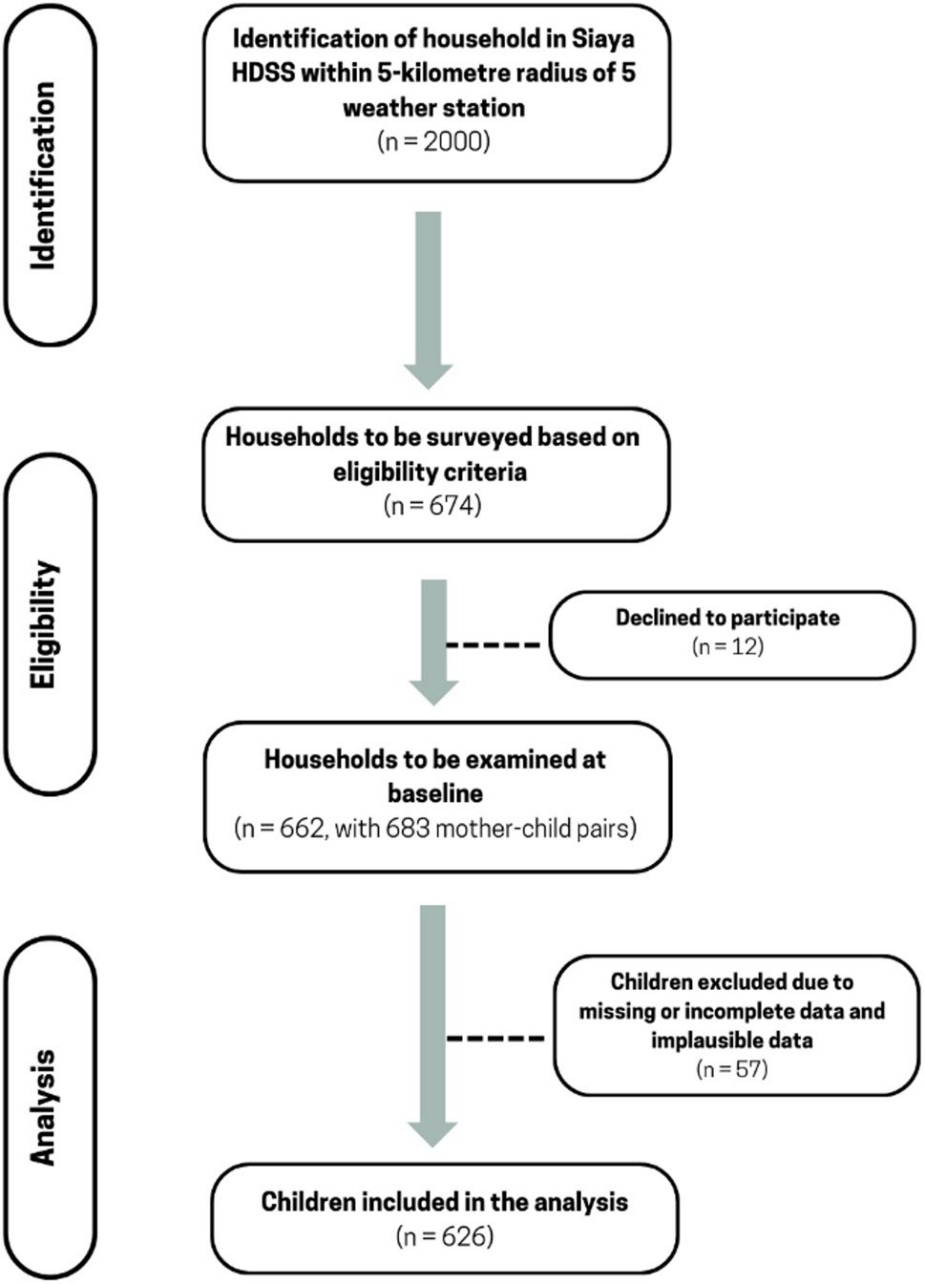
Flowchart for the recruitment of participants

Sampling and recruitment were conducted within the framework of the Health and Demographic Surveillance System (HDSS) in Siaya County. Using probability proportional to the population size (PPS), the participating households were selected from a random sample of 2000 households from 385 villages within a 5-kilometer radius of five weather stations. The inclusion criteria were having at least one child at the age of complementary feeding (6–23 months), being registered as permanent residents of the HDSS for at least 4 months before the data collection, and having access to water and land to support an intended home gardening intervention. Among those households screened for eligibility, 674 met the inclusion criteria; 12 households declined, and the final sample size was 662 households with 683 mother-child pairs (Fig. 1).

### Data collection

Anthropometric measures, including weight and height, were taken twice by trained field enumerators, to the precision of one decimal point, with a calibrated weighing scale (Seca 874, Germany) and an infantometre (Seca 417, Germany), respectively. Weight was measured with minimal clothing to reduce measurement errors. For height, we measured recumbent length if children could not stand, were below 24 months, or were shorter than 85 cm. Prior to data collection, enumerators underwent standardization exercises to assess inter-rater reliability and the technical error of measurement. The weight-for-height z-score (WHZ), weight-for-age z-score (WAZ), and height-for-age z-score (HAZ) were calculated according to the WHO reference method to determine the nutritional status of children. This means, WHZ < −2 was defined as wasting, WAZ < −2 as underweight, and HAZ < −2 as stunting.

Dietary assessment was conducted using the African-specific Food Propensity Questionnaire (AFPQ) (Supplementary File 1) to determine the habitual food intake of the children over the past 6 months. The AFPQ is an adapted version of the food propensity questionnaire developed within the European Prospective Investigation into Cancer and Nutrition (EPIC) cohort study in 2006. The tool was contextualized, pilot-tested, and refined for use in Ghanaian populations [17]. For the Kenyan context, the AFPQ was culturally adapted to ensure relevance and comprehensibility for local diets, particularly those consumed by children. The AFPQ was administered to primary caregivers and was collected digitally using the survey solution platform (HDSS 2.0 susol). In the AFPQ, 134 food items that are commonly consumed in Kenya were used to measure the frequency of standard servings of food intake. These food items were then grouped into 29 food groups based on their similarities in culinary use and nutrient profiles. The energy intakes in kcal/d and nutrient consumption in grams per day (mg/d) were calculated by applying appropriate nutrient databases (West African Food Composition Table, Kenya Food Composition Table, United States Food and Nutrition Database).

The information about covariates was obtained through the demographic and socio-economic modules of the HDSS questionnaire. Demographic information included children’s age (in months), sex, caregiver’s age (in years), and mother’s marital status. Socioeconomic data comprised the mother’s educational background, maternal occupation, household size, and the number of children under five years residing in the household.

### Statistical analysis

Statistical analysis was performed using SAS 9.4 (SAS Institute Inc., Cary, NC, USA) and STATA version 16 (STATA Corp LLC, Texas, USA). Data are presented as numbers (percentages) for categorical variables or as medians and interquartile ranges (IQR) for continuous variables. For categorical variables, chi-square tests were performed to examine differences in the undernutrition status between boys and girls. For continuous variables, Mann-Whitney tests were applied to compare the differences in other characteristics of the children across both sexes.

Dietary patterns related to climate-sensitive micronutrients were identified by RRR using the PROC PLS function in SAS 9.4. In the RRR model, 29 food groups were used as predictor variables and climate-sensitive micronutrients (standardized iron, zinc, selenium, and vitamin A) were subjected as response variables. We retained only the first dietary pattern score for the subsequent analysis, because this score explained the maximum variation in the response variables, which is the primary criterion for pattern selection in RRR. The factor loading ≥ 0.20 was selected to define important food groups. For every participant, we calculated the adherence to the dietary pattern score by summing up the standardized intake of food groups weighed by their respective factor loading. Factor loading is equivalent to a simple correlation between the food group and the extracted pattern score. For further analysis, we constructed quartiles of the RRR-derived dietary patterns. Next, we fitted multiple-adjusted linear regression analyses to calculate the associations between the dietary pattern scores and anthropometric z-scores, expressed as beta coefficients (β) and their 95% confidence intervals (CIs). The regression analyses were performed by quartiles (using the first quartile as the reference category) and per 1-score point increase. We fitted three adjustment models: Model 1 adjusted for the child’s age; Model 2 additionally adjusted for the mother’s age, education, occupation, marital status, household size, and the number of under-fives; Model 3 additionally adjusted for total energy intake (kcal/d), fibre intake (g/d), and breastfeeding status. A *p*-value < 0.05 was considered statistically significant.

## Results

In this study, the median age of mothers was 29 years (IQR: 24–34 years). The majority of mothers were married (77.3%). A substantial proportion of mothers (64.7%) had completed primary education, and the predominant occupation was subsistence farming (54.1%) (Table 1).

**Table 1:** Socio-demographic characteristics of children under 5 years of age (*N* = 626)

Characteristics		Total (*N* = 626)
Mother age (years)		29 (24, 34)
Marital status of the mother		
Single		142 (22.7)
Married		484 (77.3)
Education level of the mother		
None		3 (0.5)
Primary		394 (64.7)
Secondary		182 (29.9)
Post-secondary		30 (4.9)
Occupation of the mother		
Housewife		118 (18.9)
Subsistence farmer		339 (54.1)
Skilled workers/labourers		125 (20.0)
Business		44 (7.0)
Household size (number of people)		6 (5, 7)
Number of under-fives in a household		2 (1, 2)

Data are expressed as median (interquartile range) for continuous variables and number (percentage) for categorical variables

Boys had a significantly lower median WAZ of −0.47 (IQR: −1.31, 0.29) than girls (median WAZ: −0.20; IQR: −0.91, 0.60). Similarly, boys had a lower median WHZ and HAZ (−0.02 and − 0.88, respectively) than girls, who had a median WHZ and HAZ of 0.18 and − 0.54, respectively (*p* < 0.05) (Table 2). The corresponding proportions were for underweight 8.2%, for wasting 4.8%, and for stunting 17.9%. These numbers were higher in boys than in girls (Table 2). There were no significant differences in nutrient intakes between boys and girls.

**Table 2:** Nutritional characteristics of children under 5 years of age (*N* = 626)

Characteristics		Total (*N* = 626)	Girls (*n* = 287)	Boys (*n* = 339)	*p*-value
Age (months)		15 (11, 19)	15 (10, 19)	15 (11, 19)	0.376
Breastfeeding status, yes		463 (74.0)	223 (77.7)	240 (70.8)	0.050
Protein-energy status					
Weight-for-age (WAZ) z-score		−0.35 (−1.10, 0.47)	−0.20 (0.91, 0.60)	−0.47 (−1.31, 0.29)	0.001
Weight-for-height (WHZ) z-score		0.06 (−0.70, 0.77)	0.18 (−0.62, 0.80)	−0.02 (−0.81, 0.71)	0.043
Height-for-age (HAZ) z-score		−0.69 (−1.63, 0.18)	−0.54 (−1.47, 0.44)	−0.88 (−1.79, 0.03)	0.007
Underweight (WAZ < −2)		51 (8.2)	14 (4.9)	37 (10.9)	0.006
Wasting (WHZ < −2)		30 (4.8)	8 (2.8)	22 (6.5)	0.031
Stunting (HAZ < −2)		112 (17.9)	38 (13.2)	74 (21.8)	0.005
Macro- and micro-nutrient intake					
Total protein intake (g/day)		31.9 (22.5, 38.8)	31.1 (21.3, 38.0)	32.1 (24.0, 39.2)	0.289
Total fat intake (g/day)		27.5 (18.6, 37.3)	26.5 (17.5, 36.7)	28.7 (19.8, 38.3)	0.141
Total carbohydrate intake (g/day)		153.2 (103.8, 203.7)	151.7 (100.0, 212.4)	158.2 (108.8, 201.7)	0.642
Total energy intake (kcal/day)		1004.9 (708.8, 1333.0)	991.2 (662.2, 1364.4)	1047.5 (742.2, 1329.6)	0.478
Fiber intake (g/day)		14.7 (10.5, 18.2)	14.4 (9.8, 18.3)	14.9 (10.6, 18.1)	0.483
Iron intake (mg/day)		7.3 (5.3, 8.7)	7.0 (5.0, 8.7)	7.4 (5.4, 8.7)	0.259
Zinc intake (mg/day)		5.9 (4.1, 7.5)	6.0 (4.0, 7.4)	5.9 (4.2, 7.5)	0.843
Selenium intake (mg/day)		49.0 (31.8, 66.6)	50.1 (32.5, 68.5)	48.1 (31.4, 65.3)	0.417
Vitamin A intake (µg/day)		76.5 (44.8, 139.6)	76.8 (43.7, 130.2)	76.2 (45.4, 144.5)	0.543
ß-carotene intake (µg/day)		3848.7 (2544.9, 5385.0)	3588.8 (2483.2, 5510.1)	3990.2 (2603.1, 5336.7)	0.340
Retinol equivalent (µg/day)		432.4 (279.3, 596.2)	424.4 (273.5, 585.1)	448.5 (285.2, 598.0)	0.456
Thiamin intake (mg/day)		0.8 (0.6, 1.0)	0.8 (0.5, 0.9)	0.8 (0.6, 1.0)	0.121
Riboflavin intake (mg/day)		0.6 (0.4, 0.8)	0.6 (0.4, 0.8)	0.6 (0.4, 0.8)	0.415
Niacin intake (mg/day)		4.9 (3.5, 6.4)	4.8 (3.4, 6.5)	5.0 (3.5, 6.4)	0.509
Vitamin B6 intake (mg/day)		0.66 (0.46, 0.9)	0.6 (0.4, 0.9)	0.7 (0.5, 0.9)	0.522
Folate intake (µg/day)		150.7 (101.7, 189.6)	146.5 (99.8, 187.8)	152.5 (102.2, 190.4)	0.743
Cobalamin intake (µg/day)		2.0 (1.1, 2.9)	2.0 (1.1, 3.0)	2.0 (1.2, 2.9)	0.888
Vitamin C intake (mg/day)		35.8 (20.4, 57.9)	34.9 (20.3, 60.5)	35.9 (20.4, 55.7)	0.593
Vitamin D intake (µg/day)		2.4 (1.0, 4.3)	2.3 (1.0, 4.3)	2.4 (1.1, 4.3)	0.725
Vitamin E intake (mg/day)		5.2 (2.9, 6.9)	5.0 (2.8, 6.8)	5.3 (3.3, 7.2)	0.190

Data are expressed as medians (interquartile ranges) for continuous variables and numbers (percentages) for categorical variables

*P*-values were calculated by Mann-Whitney-U tests for continuous variables and by Chi-Square tests for categorical variables

Table 3 shows the RRR-derived dietary patterns with their factor loadings of food groups and explained variation in food intakes. The dietary patterns explained 68% and 65% of micronutrient intakes among boys and girls, respectively. The response weights showed positive directions of the relationships between the food groups and micronutrient intakes. The dietary patterns were characterized by vegetables, fish, potatoes, coffee and tea, white bread and cereals, fruits, rice and pasta, fermented food, and legumes. Among boys, red meat showed higher factor loading and roots, tubers, and eggs exhibited lower factor loadings than among girls. Characteristics of children stratified by sex across the quartile of their dietary patterns are presented in Supplementary Tables S1 and S2. Significant differences in education levels, as well as macro- and micronutrient intakes, were observed across quartiles of dietary patterns among both sexes.

**Table 3:** Factor loading and percentage variation of dietary patterns identified by reduced rank regression stratified by sexes

		Girls (*n* = 287)		Boys (*n* = 339)	
		Factor loading	Explained variance (%)	Factor loading	Explained variance (%)
Food groups					
Fruits		0.33	35.2	0.26	21.7
Vegetables		0.32	33.7	0.34	35.3
Fish		0.32	33.8	0.29	25.5
Potatoes		0.30	28.4	0.29	25.3
White bread/cereals		0.27	23.4	0.27	22.4
Rice/pasta		0.26	22.2	0.26	21.5
Other oils		0.24	19.2	0.21	13.0
Fermented food		0.24	17.8	0.25	20.1
Roots/tubers		0.23	17.6	0.13	5.3
Coffee/tea		0.23	16.4	0.28	24.0
Legumes		0.22	15.9	0.24	18.1
Eggs		0.22	15.0	0.19	11.2
Red meat		0.17	9.2	0.23	16.4
Cakes/sweets		0.16	8.2	0.15	7.4
Vegetable soups/stews		0.15	7.6	0.14	5.9
Poultry		0.14	6.1	0.16	7.6
Condiments		0.10	3.1	0.10	3.2
Whole grains/bread/cereals		0.08	2.3	0.10	3.1
Dairy products		0.08	1.8	0.09	2.4
Sodas/juices		0.07	1.8	0.13	4.8
Margarine		0.07	1.4	0.20	12.1
Nuts/seeds		0.04	0.6	0.05	0.7
Cooking fats		0.04	0.4	0.06	1.1
Processed meat		0.03	0.2	0.04	0.4
Meaty mixed dishes		0.02	0.1	−0.07	1.5
Palm oil		0.02	0.1	0.03	0.2
Sweet spreads		0.02	0.1	0.06	1.2
Vegetarian mixed dishes		0.01	0.0	−0.02	0.1
Olive oil		−0.01	0.1	0.01	0.0
Total explained variation in food groups (%)			11.1		10.7
Response weights					
Iron		0.56		0.55	
Selenium		0.56		0.55	
Zinc		0.38		0.42	
Vitamin A		0.48		0.45	
Explained variation in response variables (%)					
Iron			81.3		83.4
Selenium			37.7		46.9
Zinc			81.4		82.6
Vitamin A			61.0		59.9
Total explained variation in micronutrient intakes (%)			65.4		68.2

Factor loadings are correlations between food groups and dietary pattern scores. Factor loading cut-off ≥ 0.20 was used to determine the food groups that related to the pattern.

Table 4 shows the associations between RRR-derived dietary patterns and children’s WAZ, WHZ, and HAZ. Among boys, the dietary pattern showed no linear association with any of the anthropometric z-scores. In contrast, among girls, there were positive associations of dietary pattern adherence with WAZ and WHZ. This was seen across pattern-quartiles and per 1 score-point increase and remained stable in the fully adjusted model 3 (WAZ: β = 0.19; 95% CI: 0.02, 0.36; WHZ: β = 0.16; 95% CI: −0.01, 0.34). However, the dietary pattern was not associated with HAZ. Additionally, sensitivity analysis with further adjustment for morbidity (diarrhea and fever in the past four weeks) showed that the associations remained consistent in both, direction and magnitude across both sexes (Supplementary Table S3).

**Table 4:** Associations between dietary pattern scores and anthropometric z-scores among girls and boys

Outcome	*N*	Crude		Model 1		Model 2		Model 3	
		ß (95% CI)	*p*-value	ß (95% CI)	*p*-value	ß (95% CI)	*p*-value	ß (95% CI)	*p*-value
Girls (*n* = 287)									
Weight-for-age (WAZ) z-score									
Per 1 score-point increase		0.00 (−0.08, 0.08)	0.939	0.05 (−0.03, 0.14)	0.213	0.07 (−0.02, 0.16)	0.129	0.19 (0.02, 0.36)	0.033
Quartile 1	72	Ref							
Quartile 2	72	0.28 (−0.10, 0.67)	0.146	0.43 (0.05, 0.82)	0.028	0.52 (0.12, 0.92)	0.011	0.68 (0.24, 1.12)	0.003
Quartile 3	72	0.19 (−0.19, 0.58)	0.323	0.37 (−0.02, 0.76)	0.060	0.44 (0.04, 0.84)	0.030	0.69 (0.16, 1.21)	0.010
Quartile 4	71	0.12 (−0.26, 0.51)	0.536	0.37 (−0.03, 0.77)	0.072	0.41 (0.00, 0.82)	0.049	0.86 (0.13, 1.58)	0.021
*p-value for trend*		0.520		0.123		0.055		0.022	
Weight-for-height (WHZ) z-score									
Per 1 score-point increase		0.02 (−0.06, 0.10)	0.627	0.01 (−0.08, 0.09)	0.842	0.02 (−0.07, 0.10)	0.728	0.16 (−0.01, 0.34)	0.071
Quartile 1	72	Ref							
Quartile 2	72	0.25 (−0.13, 0.63)	0.202	0.23 (−0.16, 0.62)	0.248	0.31 (−0.09, 0.72)	0.130	0.54 (0.09, 0.98)	0.019
Quartile 3	72	0.33 (−0.04, 0.72)	0.085	0.31 (−0.08, 0.71)	0.119	0.39 (−0.02, 0.79)	0.061	0.76 (0.23, 1.29)	0.005
Quartile 4	71	0.17 (−0.21, 0.56)	0.374	0.14 (−0.26, 0.55)	0.489	0.17 (−0.25, 0.58)	0.431	0.83 (0.10, 1.57)	0.027
*p-value for trend*		0.358		0.440		0.251		0.040	
Height-for-age (HAZ) z-score									
Per 1 score-point increase		−0.06 (−0.16, 0.04)	0.257	0.07 (−0.03, 0.17)	0.152	0.09 (−0.01, 0.19)	0.079	0.14 (−0.06, 0.35)	0.167
Quartile 1	72	Ref							
Quartile 2	72	0.10 (−0.38, 0.58)	0.679	0.45 (0.00, 0.90)	0.048	0.52 (0.06, 0.99)	0.028	0.53 (0.01, 1.05)	0.047
Quartile 3	72	−0.23 (−0.71, 0.24)	0.337	0.20 (−0.26, 0.65)	0.396	0.24 (−0.23, 0.70)	0.317	0.22 (−0.40, 0.83)	0.490
Quartile 4	71	−0.18 (−0.66, 0.30)	0.451	0.41 (−0.06, 0.88)	0.088	0.47 (−0.01, 0.95)	0.057	0.44 (−0.41, 1.30)	0.312
*p-value for trend*		0.479		0.185		0.113		0.196	
Boys (*n* = 339)									
Weight-for-age (WAZ) z-score									
Per 1 score-point increase		−0.08 (−0.17, 0.01)	0.092	−0.03 (−0.13, 0.07)	0.550	−0.04 (−0.14, 0.06)	0.433	−0.06 (−0.23, 0.11)	0.508
Quartile 1	85	Ref							
Quartile 2	85	−0.08 (−0.48, 0.32)	0.694	0.06 (−0.35, 0.47)	0.776	0.04 (−0.37, 0.45)	0.852	0.09 (−0.39, 0.56)	0.718
Quartile 3	85	−0.21 (−0.61, 0.20)	0.315	−0.06 (−0.47, 0.35)	0.775	−0.13 (−0.54, 0.28)	0.532	−0.15 (−0.68, 0.37)	0.575
Quartile 4	84	−0.31 (−0.71, 0.09)	0.132	−0.10 (−0.53, 0.32)	0.632	−0.13 (−0.56, 0.30)	0.545	−0.16 (−0.85, 0.53)	0.648
*p-value for trend*		0.449		0.870		0.777		0.674	
Weight-for-height (WHZ) z-score									
Per 1 score-point increase		−0.08 (−0.17, 0.01)	0.071	−0.09 (−0.19, 0.00)	0.052	−0.09 (−0.19, 0.00)	0.058	−0.10 (−0.27, 0.07)	0.262
Quartile 1	85	Ref							
Quartile 2	85	−0.10 (−0.48, 0.27)	0.588	−0.13 (−0.53, 0.27)	0.499	−0.15 (−0.56, 0.24)	0.435	−0.10 (−0.57, 0.36)	0.671
Quartile 3	85	−0.22 (−0.60, 0.16)	0.259	−0.25 (−0.64, 0.14)	0.212	−0.29 (−0.69, 1.11)	0.152	−0.25 (−0.77, 0.27)	0.340
Quartile 4	84	−0.33 (−0.71, 0.05)	0.091	−0.37 (−0.78, 0.03)	0.072	−0.37 (−0.78, 0.05)	0.085	−0.30 (−0.98, 0.37)	0.377
*p-value for trend*		0.362		0.309		0.323		0.765	
Height-for-age (HAZ) z-score									
Per 1 score-point increase		−0.05 (−0.17, 0.06)	0.352	0.08 (−0.04, 0.19)	0.185	0.06 (−0.06, 0.17)	0.331	0.02 (−0.19, 0.22)	0.885
Quartile 1	85	Ref							
Quartile 2	85	−0.05 (−0.54, 0.44)	0.831	0.30 (−0.17, 0.78)	0.211	0.30 (−0.18, 0.78)	0.217	0.28 (−0.28, 0.83)	0.325
Quartile 3	85	−0.10 (−0.59, 0.38)	0.673	0.27 (−0.21, 0.75)	0.271	0.19 (−0.29, 0.67)	0.437	0.05 (−0.56, 0.67)	0.868
Quartile 4	84	−0.21 (−0.70, 0.28)	0.397	0.31 (−0.18, 0.81)	0.211	0.25 (−0.25, 0.75)	0.332	0.03 (−0.77, 0.84)	0.934
*p-value for trend*		0.853		0.546		0.646		0.631	

The beta coefficients and 95% confidence intervals (CIs) were calculated by multiple-adjusted linear regression

Model 1: adjusted for age

Model 2: model 1 + mother’s age, education, occupation, marital status, number of children under five, and household size

Model 3: model 2 + total energy intake (kcal/day), fibre intake (g/d), and breastfeeding status

## Discussion

This study among young children in rural Kenya derived dietary patterns related to climate-sensitive micronutrients and established their associations with nutritional status according to anthropometric measurements. We found that boys had lower mean WAZ, WHZ, and HAZ than girls. Dietary patterns derived from RRR analysis were characterized by high consumption of vegetables, fish, potatoes, coffee and tea, white bread and cereals, fruits, rice and pasta, fermented food, and legumes. Minor differences were observed between sexes, with red meat being featured among boys, while roots, tubers, and eggs were more prominent among girls. Only among girls, these dietary patterns were positively associated with anthropometric markers of general and acute undernutrition (WAZ and WHZ). These findings may reflect the differing temporal sensitivity of these anthropometric indicators. WAZ and WHZ capture acute or short-term nutritional status and respond relatively quickly to improvements in energy and nutrient intake. In contrast, HAZ reflects chronic undernutrition and cumulative growth faltering over a longer period, often starting in utero and early infancy [2]. Consequently, current dietary intake among children aged 6–23 months may not be sufficient to reverse pre-existing linear growth deficits, and the cross-sectional study design could not confirm these findings.

The sex difference in undernutrition outcomes is consistent with previous research, which documented that boys exhibit poorer growth outcomes compared to girls, with lower mean WAZ and HAZ in early childhood in several low- and middle-income countries [18, 19], including Ghana [20–22] and Tanzania [23]. Similarly, a systematic review of sex differences in stunting among under-fives in sub-Saharan African countries reported that boys are consistently more likely to have stunting than girls [24]. However, a previous study conducted in Eastern Kenya found contrary results. This cross-sectional study in Ukambani, conducted in 2008, reported that girls were more likely to have stunting, underweight, and wasting than boys [25]. The observed sex differences may be attributed to several factors, involving a complex interplay of biological, social, economic, and cultural factors that influence feeding and care practices [26]. For instance, boys may have higher metabolic demands and are more vulnerable to environmental stressors in early life, making them more susceptible to undernutrition when nutrient intake is inadequate [24, 26]. Notably, a recent study reported that the disparity in growth patterns between boys and girls varies with age. Boys show more severe growth failure in the first 30 months, but this gap narrow as they grow older [19]. Additionally, societal norms may influence or dictate different dietary and feeding practices, as well as food choices, for boys and girls. Another hypothesis also suggests that daughter preference arose as a consequence of reduced socioeconomic conditions [24]. Nevertheless, conflicting studies suggest that a higher social value is placed on sons at the expense of daughters [27], including instances of dietary discrimination [28]. These findings contradict the notion that female children hold a nutritionally privileged position compared to their male counterparts. Further research is therefore needed to confirm and/or provide an explanation, including a biological explanation, of sex differences in child undernutrition across socio-economic strata.

The dietary patterns identified in the study showed similar characteristics across both sexes, characterized by positive loadings for both animal- and plant-source protein, which are essential for a child’s growth and development. However, the dietary pattern observed in girls included roots or tubers and eggs, a feature not evident in boys. Roots and tubers, such as sweet potatoes and cassava, are significant sources of complex carbohydrates, primarily in the form of starch. The accumulation of starch in tuberous roots is vital for energy provision, which is crucial for the rapid growth phases typical in early childhood [29, 30]. The high carbohydrate and fibre content, as well as the presence of iron, thiamine, and folate in these food groups, not only support energy needs but also contribute to overall caloric intake, energy metabolism, and digestive health. All of these are important for preventing undernutrition in young children and further promoting growth [30, 31]. Eggs are particularly noteworthy for their high-quality protein content and essential fatty acids, which have been demonstrated to significantly enhance plasma iron levels and transferrin saturation, crucial factors for development and overall health in young children [32]. Eggs contain healthy fats such as phospholipids and PUFAs that play a crucial role in the growth of infants and young children [33]. Eggs are also rich in protein and micronutrients, such as vitamins B12, A, riboflavin, B5, D, as well as choline, selenium, and other critical nutrients at levels above or comparable to those found in other animal-source foods. These play vital roles in the metabolic processes, growth, and various body functions [32, 34–37]. A recent systematic review and meta-analysis highlights that complementary feeding, particularly based on eggs, has a beneficial effect on improving physical growth, including HAZ and WAZ [33].

Noticeably, boys’ dietary patterns featured the red meat food group, but not roots, tubers, and eggs. While certain studies have demonstrated significant benefits of meat consumption as part of the dietary habit for growth among young children in LMICs, others report mixed results [38, 39]. We found no association between dietary patterns featuring red meat and growth outcomes. One plausible explanation for the lack of association among boys found in our study could stem from low overall dietary diversity [40]. Studies have shown that consuming a diverse range of foods is a crucial factor for healthy nutritional status and growth outcomes among young children [41–43]. Indeed, the synergistic effect of combining a large variety of foods in an infant’s complementary feeding practice can significantly enhance nutrient intake and adequacy [44], thus preventing undernutrition and promoting healthy growth [45]. For instance, the protein, fat, and micronutrients from eggs, meat, and fish can complement the carbohydrates and fibre from tubers, fermented food, and legumes, providing a diverse nutritional profile that supports energy needs and growth during this critical developmental stage. Studies have also emphasized that children who consume a variety of foods tend to have better nutritional outcomes [20, 46]. Taken together, the findings suggest that consuming many different types of food groups as part of the complementary feeding practice enhances dietary diversity, nutrient adequacy, and nutrient density, thereby leading to better nutritional and growth outcomes. The strengths of this study include the innovative approach of using RRR to derive dietary patterns related to climate-sensitive micronutrient, which provide valuable insights into the relationships between food intake, nutrient supply, and nutritional status. The focus on iron, zinc, selenium, and vitamin A as response variables is particularly relevant, given the critical role these micronutrients play in child growth and development, and their potential vulnerability to climate change impacts. The limitations of our study are fourfold. First, dietary habits were measured by a self-reported questionnaire; thus, recall bias and measurement errors in reporting dietary intake are inevitable. However, in population-based studies, the tool is useful to rank participants according to their intakes. Second, the cross-sectional nature of our study cannot infer causality. Yet, biologically, it is more plausible that food intake influences nutritional status than vice versa. Third, although the RRR-derived dietary patterns related to climate-sensitive micronutrients explained a substantial amount of the variations in response variables, the predictors (food groups) and responses (nutrients) originated from the same dietary assessment tool. This may have violated the independence assumption for response variables and may have created multicollinearity. Moreover, response variables in the RRR method may differ in other studies, making it difficult to compare the dietary pattern reported in other studies directly with our findings. Fourth, we might have faced the potential of residual and unmeasured confounding in the regression models.

Our findings align with the growing body of evidence that climate variability is already impacting food and nutrition security in Kenya. The observed dietary patterns—rich in vegetables and legumes—reflect food groups whose production and availability are particularly vulnerable to rising temperatures, recurrent droughts, and carbon emissions. Consequently, promoting diversified diets that integrate both climate-resilient crops and sustainable sources of aquatic protein is critical to safeguarding child nutrition under ongoing climate change. In practical terms, the study therefore offers locally relevant evidence to inform Kenya’s Climate-Smart Agriculture Implementation Framework and National Food and Nutrition Security Policy, both of which emphasize dietary diversification and climate-resilient production as adaptation priorities to maintain access to bioavailable micronutrients for young children. Taken together, from a public health perspective, our findings suggest several key strategies. First, our study underscores the importance of promoting diverse and nutrient-rich diets to improve child nutrition. Second, gender-sensitive interventions should be tailored to address the specific needs of boys and girls, given the observed sex differences in nutritional outcomes. Third, the implementation of nutrition education programs, particularly designed for primary caregivers, that are tailored to local contexts to enhance nutrient adequacy and support healthy growth in young children, might be beneficial.

## Conclusion

To conclude, in this cross-sectional study among children aged 6–23 months living in Siaya County, Kenya, RRR-derived dietary patterns that are related to climate-sensitive micronutrients are associated with better nutritional status according to anthropometric measurements, but only among girls and only with markers of general and acute undernutrition. The diverse composition of these dietary patterns, including plant-based and animal-based protein sources, highlights the importance of promoting diverse diets that are rich in essential micronutrients. These strategies will contribute to combating undernutrition in this vulnerable population, particularly in the context of climate change.

## Supplementary Information

Supplementary Material 1

Supplementary Material 2

## Data Availability

The datasets generated and/or analyzed during the current study are not publicly available and are restricted for research use only. Datasets are available from the corresponding author on reasonable request.
