# Supplementary material for: Reduced rank regression-derived dietary patterns related to climate-sensitive micronutrients and their associations with child undernutrition among young children in rural Kenya: findings from the ALIMUS study
Source: BMC Public Health. 2026 Jan 13;26:348. doi: 10.1186/s12889-026-26265-z (PMC12849572; doi:10.1186/s12889-026-26265-z)
Supplement: Supplementary file 1 — Supplementary Material 1 [file 12889_2026_26265_MOESM1_ESM.pdf]

African-Specific Food Propensity Questionnaire

"Climate-sensitive nutrients, nutritional status and health in sub-Saharan Africa"

A study located in Kisumu, Kenya, and Nouna, Burkina Faso

Funded by the DFG and Bosch Foundation

Original drafted by Ina Danquah

Amended by Gabriele Stieglbauer, Erick Agure, and Isabel Mank

Heidelberg Institute of Global Health (HIGH), INF 324, 69120 Heidelberg

Last updated: 11.03.2020, 20.06.2021

Modified according to the Ghana-Specific Food Propensity Questionnaire of the Research on Obesity and Diabetes among African Migrants (RODAM)-Study

Introduction to the questionnaire

What we eat and how much we eat is important for our health. This is especially the case for children under the age of 5 years, who need a healthy and nutritious diet to grow well. Therefore, we like to ask you some additional questions to learn more about the eating habits of your child. With those findings, we are able to understand the aspects of your child's diet that contribute to his/ her health. This way we will be able to develop health advice that is specific to young children living in rural sub-Saharan Africa and, ultimately, improves the health of your community.

I will now list a number of food items. I would like to find out whether your child ate these food items over **the past 6 months, and if yes, how often on average**. For each food item, there are distinct response categories that I will read out for you.

Start time ………………………………………………… (hh:mm) **(For interviewers only)**

………

Before we begin with the interview, please, indicate how often your child consumed breast milk **<u>yesterday</u>**.

**Mother's milk (breast milk)**	◻ never	◻ 4-5 times a day
	◻ once a day	◻ 6-7 times a day
	◻ 2-3 times a day	◻ More than 7 times a day

Bread and Milk Products

Now, please indicate your child's average intake of the following food items during the past 6 months. I will start listing the food items with bread and milk products.

Bread and Cereals

Bread and bread-like products (e.g. Mandazi, chapati) (one slice or piece)

- □ never
- □ 6 times per week or less frequent
- □ 1-2 times a day
- □ 3-4 times a day
- □ 5-6 times a day
- □ 7-8 times a day
- □ 9 times a day or more frequent

Muesli, cereals, cornflakes, Cold porridge (3 soup spoons/1cup)

- □ never
- □ once a month or less frequent
- □ 2-3 times a month
- □ 1-2 times a week
- □ 3-4 times a week
- □ 5-6 times a week
- □ 1 time a day or more frequent

Hot cereal, hot porridge (e.g. tô out of millet or sorghum), oats (3 soup spoons/1cup)

- □ never
- □ once a month or less frequent
- □ 2-3 times a month
- □ 1-2 times a week
- □ 3-4 times a week
- □ 5-6 times a week
- □ 1 time a day or more frequent

Bread variety

Rye-, Multigrain bread and buns	□ never	□ rarely	□ sometimes	□ frequently	□ always
Whole grain bread and buns	□ never	□ rarely	□ sometimes	□ frequently	□ always
White, wheat bread, buns, toast, sugar bread	□ never	□ rarely	□ sometimes	□ frequently	□ always

Spreads

Butter on bread	□ never	□ rarely	□ sometimes	□ frequently	□ always
Regular margarine	□ never	□ rarely	□ sometimes	□ frequently	□ always
Fat-reduced margarine	□ never	□ rarely	□ sometimes	□ frequently	□ always
Peanut butter	□ never	□ rarely	□ sometimes	□ frequently	□ always

Marmalade, jam, jelly, honey	□ never	□ 3-4 times a week
(1 teaspoon)	□ once a month or less frequent	□ 5-6 times a week
	□ 2-3 times a month	□ 1-2 times a day
	□ 1-2 times a week	□ 3 times a day or more frequent

Milk and Yoghurt

Cocoa, chocolate milk, fruit milk	◻ never	◻ 3-4 times a week
(5.3 oz or 150 ml/ half a water glass)	◻ once a month or less frequent	◻ 5-6 times a week
	◻ 2-3 times a month	◻ 1 time a day or more frequent
	◻ 1-2 times a week	
Animal milk, powdered milk	◻ never	◻ 3-4 times a week
(5.3 oz or 150 ml/ half a water glass)	◻ once a month or less frequent	◻ 5-6 times a week
	◻ 2-3 times a month	◻ 1-2 times a day
	◻ 1-2 times a week	◻ 3 times a day or more frequent
Plain yoghurt, buttermilk	◻ never	◻ 3-4 times a week
(5.3 oz or 150 ml)	◻ once a month or less frequent	◻ 5-6 times a week
	◻ 2-3 times a month	◻ 1 time a day or more frequent
	◻ 1-2 times a week	
Sour milk, kefir, fermented milks	◻ never	◻ 3-4 times a week
(5.3 oz or 150 ml/ half a water glass)	◻ once a month or less frequent	◻ 5-6 times a week
	◻ 2-3 times a month	◻ 1 time a day or more frequent
	◻ 1-2 times a week	

Flavoured yoghurt (5.3 oz or 150 ml)	◻ never	◻ 3-4 times a week
	◻ once a month or less frequent	◻ 5-6 times a week
	◻ 2-3 times a month	◻ 1 time a day or more frequent
	◻ 1-2 times a week	

Greek Yoghurt and Cheese

[Greek yoghurt (curd), Greek yoghurt with herbs (no fruit quark) (1 tablespoon)]	◻ never	◻ 3-4 times a week
	◻ once a month or less frequent	◻ 5-6 times a week
	◻ 2-3 times a month	◻ 1 time a day or more frequent
	◻ 1-2 times a week	
Cream cheese, sour cream (full fat) (1 tablespoon)	◻ never	◻ 3-4 times a week
	◻ once a month or less frequent	◻ 5-6 times a week
	◻ 2-3 times a month	◻ 1 time a day or more frequent
	◻ 1-2 times a week	
Soft cheese (e.g. Camembert, Brie, Munster, Burgos type) (one portion)	◻ never	◻ 3-4 times a week
	◻ once a month or less frequent	◻ 5-6 times a week
	◻ 2-3 times a month	◻ 1 time a day or more frequent
	◻ 1-2 times a week	

Semi-soft and firm cheese (e.g. Gouda, Emmental, or own production) (one portion)	◻ never	◻ 3-4 times a week
	◻ once a month or less frequent	◻ 5-6 times a week
	◻ 2-3 times a month	◻ 1 time a day or more frequent
	◻ 1-2 times a week	

Fruits and Vegetables

Please indicate your child's average intake of the following food items during the past 6 months. Please treat foods with a specific harvesting season as if they were available throughout the year!

Fruits

Apple, pear, fresh (whole)	◻ never	◻ 3-4 times a week
	◻ once a month or less frequent	◻ 5-6 times a week
	◻ 2-3 times a month	◻ 1-2 times a day
	◻ 1-2 times a week	◻ 3 times a day or more frequent
Orange, mandarin orange, kiwi, fresh (whole)	◻ never	◻ 3-4 times a week
	◻ once a month or less frequent	◻ 5-6 times a week
	◻ 2-3 times a month	◻ 1-2 times a day
	◻ 1-2 times a week	◻ 3 times a day or more frequent

Banana (whole)	□ never<br>□ once a month or less frequent<br>□ 2-3 times a month<br>□ 1-2 times a week	□ 3-4 times a week<br>□ 5-6 times a week<br>□ 1-2 times a day<br>□ 3 times a day or more frequent
Plum, peach, apricot, nectarine, flat peach, fresh (a handful)	□ never<br>□ once a month or less frequent<br>□ 2-3 times a month<br>□ 1-2 times a week	□ 3-4 times a week<br>□ 5-6 times a week<br>□ 1-2 times a day<br>□ 3 times a day or more frequent
Strawberries, cherries (a handful)	□ never<br>□ once a month or less frequent<br>□ 2-3 times a month<br>□ 1-2 times a week	□ 3-4 times a week<br>□ 5-6 times a week<br>□ 1-2 times a day<br>□ 3 times a day or more frequent
Water melon, melon, guava (1 piece, 200 g)	□ never<br>□ once a month or less frequent<br>□ 2-3 times a month<br>□ 1-2 times a week	□ 3-4 times a week<br>□ 5-6 times a week<br>□ 1-2 times a day<br>□ 3 times a day or more frequent
Mango, papaya, pineapple, passion fruit (1 piece, 200 g)	□ never<br>□ once a month or less frequent<br>□ 2-3 times a month<br>□ 1-2 times a week	□ 3-4 times a week<br>□ 5-6 times a week<br>□ 1-2 times a day<br>□ 3 times a day or more frequent

Red and black currants, blackberries, blueberries, tree tomato (tamarillo) (a handful)	◻ never<br>◻ once a month or less frequent<br>◻ 2-3 times a month<br>◻ 1-2 times a week	◻ 3-4 times a week<br>◻ 5-6 times a week<br>◻ 1-2 times a day<br>◻ 3 times a day or more frequent
Grapes (a handful)	◻ never<br>◻ once a month or less frequent<br>◻ 2-3 times a month<br>◻ 1-2 times a week	◻ 3-4 times a week<br>◻ 5-6 times a week<br>◻ 1-2 times a day<br>◻ 3 times a day or more frequent
Stewed fruit, canned fruit (bought from market) (1 cup)	◻ never<br>◻ once a month or less frequent<br>◻ 2-3 times a month	◻ 1-2 times a week<br>◻ 3 times a week or more frequent

Dried Fruits and Nuts

Dried fruit (e.g. prunes, figs, raisins, dates) (a handful)	◻ never<br>◻ once a month or less frequent<br>◻ 2-3 times a month	◻ 1-2 times a week<br>◻ 3-4 times a week<br>◻ 5 times a week or more frequent
Nuts (salted, fresh, e.g. peanuts/groundnuts, tiger nuts, hazelnuts, walnuts, almonds, pine nuts, cashew, avocado) (a handful)	◻ never<br>◻ once a month or less frequent<br>◻ 2-3 times a month<br>◻ 1-2 times a week	◻ 3-4 times a week<br>◻ 5-6 times a week<br>◻ 1 time a day or more frequent
Seeds (e.g. pumpkin seeds/agushie, flaxseed, sesame) (1 tablespoon)	◻ never<br>◻ once a month or less frequent<br>◻ 2-3 times a month<br>◻ 1-2 times a week	◻ 3-4 times a week<br>◻ 5-6 times a week<br>◻ 1 time a day or more frequent

Plantain, Roots and Tubers

Plantain (e.g. boiled, roasted) (3 pieces, finger size)	◻ never<br>◻ once a month or less frequent<br>◻ 2-3 times a month<br>◻ 1-2 times a week	◻ 3-4 times a week<br>◻ 5-6 times a week<br>◻ 1-2 times a day<br>◻ 3 times a day or more frequent

Cassava (e.g. boiled, roasted, dried etc.) (3 pieces/slices)	◻ never<br>◻ once a month or less frequent<br>◻ 2-3 times a month<br>◻ 1-2 times a week	◻ 3-4 times a week<br>◻ 5-6 times a week<br>◻ 1-2 times a day<br>◻ 3 times a day or more frequent
Yam, cocoyam (e.g. boiled, roasted, dried etc.) (3 pieces/slices)	◻ never<br>◻ once a month or less frequent<br>◻ 2-3 times a month<br>◻ 1-2 times a week	◻ 3-4 times a week<br>◻ 5-6 times a week<br>◻ 1-2 times a day<br>◻ 3 times a day or more frequent

Potatoes

Sweet potatoes (e.g. boiled, roasted) (3 egg-sized pieces)	◻ never<br>◻ once a month or less frequent<br>◻ 2-3 times a month<br>◻ 1-2 times a week	◻ 3-4 times a week<br>◻ 5-6 times a week<br>◻ 1-2 times a day<br>◻ 3 times a day or more frequent
Potatoes (e.g. boiled, mashed, dumplings, oven baked etc.) (3 egg-sized pieces)	◻ never<br>◻ once a month or less frequent<br>◻ 2-3 times a month<br>◻ 1-2 times a week	◻ 3-4 times a week<br>◻ 5-6 times a week<br>◻ 1 time a day or more frequent

Pan-fried potatoes, French fries fried plantain/cassava/yam (one portion, 10 sticks)	◻ never<br>◻ once a month or less frequent<br>◻ 2-3 times a month	◻ 1-2 times a week<br>◻ 3 times a week or more frequent

Pounded plantain, roots, tubers or (fermented) maize

Plantain-based dough (cooked and pounded) (large orange size)	◻ never<br>◻ once a month or less frequent<br>◻ 2-3 times a month<br>◻ 1-2 times a week	◻ 3-4 times a week<br>◻ 5-6 times a week<br>◻ 1-2 times a day<br>◻ 3 times a day or more frequent
Dough from boiled maize flour- ugali (large orange size)	◻ never<br>◻ once a month or less frequent<br>◻ 2-3 times a month<br>◻ 1-2 times a week	◻ 3-4 times a week<br>◻ 5-6 times a week<br>◻ 1-2 times a day<br>◻ 3 times a day or more frequent
Dough from boiled fermented maize (large orange size)	◻ never<br>◻ once a month or less frequent<br>◻ 2-3 times a month<br>◻ 1-2 times a week	◻ 3-4 times a week<br>◻ 5-6 times a week<br>◻ 1-2 times a day<br>◻ 3 times a day or more frequent

Raw Vegetables

Carrots, pumpkin, squash (raw or cooked) (1 carrot or one portion)	◻ never<br>◻ once a month or less frequent<br>◻ 2-3 times a month<br>◻ 1-2 times a week	◻ 3-4 times a week<br>◻ 5-6 times a week<br>◻ 1 time a day or more frequent
Tomatoes, raw (whole)	◻ never<br>◻ once a month or less frequent<br>◻ 2-3 times a month<br>◻ 1-2 times a week	◻ 3-4 times a week<br>◻ 5-6 times a week<br>◻ 1 time a day or more frequent
Light and dark green lettuce, endive, chicory, Napa/Chinese cabbage, white/green cabbage, raw (one portion/ 1/3 of a plate)	◻ never<br>◻ once a month or less frequent<br>◻ 2-3 times a month<br>◻ 1-2 times a week	◻ 3-4 times a week<br>◻ 5-6 times a week<br>◻ 1 time a day or more frequent
Cucumber (one portion, 5 slices)	◻ never<br>◻ once a month or less frequent<br>◻ 2-3 times a month<br>◻ 1-2 times a week	◻ 3-4 times a week<br>◻ 5-6 times a week<br>◻ 1 time a day or more frequent

Sweet peppers, raw (whole)	□ never<br>□ once a month or less frequent<br>□ 2-3 times a month<br>□ 1-2 times a week	□ 3-4 times a week<br>□ 5-6 times a week<br>□ 1 time a day or more frequent

Garlic (1 clove)	Raw	□ no □ sometimes □ yes
	Cooked, roasted, pickled	□ no □ sometimes □ yes
Onions or onion leaves (a quarter)	Raw	□ no □ sometimes □ yes
	Cooked, roasted, pickled	□ no □ sometimes □ yes

Cooked Vegetables: 1 portion refers to 1/3 of a plate

Green leaves, spinach, Swiss chard, kale (one portion), (e.g cassava leaves, cowpea leaves, sweet potato leaves, baobab leaves, drumstick leaves, etc.)	□ never<br>□ once a month or less frequent<br>□ 2-3 times a month	□ 1-2 times a week<br>□ 3 times a week or more frequent
Cooked white/green cabbage, cauliflower, broccoli, Brussels sprouts, collard greens (one portion)	□ never<br>□ once a month or less frequent<br>□ 2-3 times a month	□ 1-2 times a week<br>□ 3 times a week or more frequent

Eggplant, garden egg, squash, zucchini, courgette, okra (one portion)	◻ never<br>◻ once a month or less frequent<br>◻ 2-3 times a month	◻ 1-2 times a week<br>◻ 3 times a week or more frequent
Legumes (e.g. cow peas, beans, chick peas, lentils) (one portion)	◻ never<br>◻ once a month or less frequent<br>◻ 2-3 times a month	◻ 1-2 times a week<br>◻ 3 times a week or more frequent
Green beans, French beans (one portion)	◻ never<br>◻ once a month or less frequent<br>◻ 2-3 times a month	◻ 1-2 times a week<br>◻ 3 times a week or more frequent
Maize corn, grilled (one corn)	◻ never<br>◻ once a month or less frequent<br>◻ 2-3 times a month	◻ 1-2 times a week<br>◻ 3 times a week or more frequent

Vegetables Soups, Stews and Sauces

Groundnut soup (3 soup ladles)	◻ never<br>◻ once a month or less frequent<br>◻ 2-3 times a month	◻ 1-2 times a week<br>◻ 3 times a week or more frequent

Palm nut soup (3 soup ladles)	◻ never<br>◻ once a month or less frequent<br>◻ 2-3 times a month	◻ 1-2 times a week<br>◻ 3 times a week or more frequent
Green-leafy stew, sauce from cocoyam/cassava leaves (3 soup spoons)	◻ never<br>◻ once a month or less frequent<br>◻ 2-3 times a month	◻ 1-2 times a week<br>◻ 3 times a week or more frequent
Tomato sauce, tomato stew (including canned/tinned tomato, crushed tomato) (3 soup ladles)	◻ never<br>◻ once a month or less frequent<br>◻ 2-3 times a month	◻ 1-2 times a week<br>◻ 3 times a week or more frequent
Lentil-, Pea-, bean soup (3 soup ladles)	◻ never<br>◻ once a month or less frequent<br>◻ 2-3 times a month	◻ 1-2 times a week<br>◻ 3 times a week or more frequent
Vegetable soup, light soup (3 soup ladles)	◻ never<br>◻ once a month or less frequent<br>◻ 2-3 times a month	◻ 1-2 times a week<br>◻ 3 times a week or more frequent

Rice, Pasta and Egg

Please indicate your child's average intake of the following food items during the past 6 months.

Rice (e.g. plain rice, fried rice, pilau)<br>(one portion/ half a cup)	◻ never<br>◻ once a month or less frequent<br>◻ 2-3 times a month<br>◻ 1-2 times a week	◻ 3-4 times a week<br>◻ 5-6 times a week<br>◻ 1 time a day or more frequent
Other grains (millet, couscous, fonio, polenta, sorghum, spelt, barley)<br>(one portion/ half a cup)	◻ never<br>◻ once a month or less frequent<br>◻ 2-3 times a month<br>◻ 1-2 times a week	◻ 3-4 times a week<br>◻ 5-6 times a week<br>◻ 1 time a day or more frequent
Pasta, noodles, macaroni<br>(one portion/ 1/3 of a plate)	◻ never<br>◻ once a month or less frequent<br>◻ 2-3 times a month	◻ 1-2 times a week<br>◻ 3-4 times a week<br>◻ 5 times a week or more frequent
Eggs, cooked or fried, omelette (whole)<br>(from chicken and guinea fowl)	◻ never<br>◻ once a month or less frequent<br>◻ 2-3 times a month<br>◻ 1-2 times a week	◻ 3-4 times a week<br>◻ 5-6 times a week<br>◻ 1 time a day or more frequent

Meat and Fish

Please indicate your child's average intake of the following food items during the past 6 months. Please note: If the respondent is unfamiliar with certain food items, still do not leave blanks, rather consider these items as "never" consumed!

Meat and meat products

Beef (one portion, 100g/ 2 soup ladles)	◻ never<br>◻ once a month or less frequent<br>◻ 2-3 times a month	◻ 1-2 times a week<br>◻ 3 times a week or more frequent
Poultry (one portion, 100g/ 2 soup ladles)	◻ never<br>◻ once a month or less frequent<br>◻ 2-3 times a month	◻ 1-2 times a week<br>◻ 3 times a week or more frequent
Goat (one portion, 100g/ 2 soup ladles)	◻ never<br>◻ once a month or less frequent<br>◻ 2-3 times a month	◻ 1-2 times a week<br>◻ 3 times a week or more frequent
Pork (one portion, 100g/ 2 soup ladles)	◻ never<br>◻ once a month or less frequent<br>◻ 2-3 times a month	◻ 1-2 times a week<br>◻ 3 times a week or more frequent
Bush meat, venison/game, caterpillar (one portion, 100g/ 2 soup ladles)	◻ never<br>◻ once a month or less frequent<br>◻ 2-3 times a month	◻ 1-2 times a week<br>◻ 3 times a week or more frequent

Intestinal/organ meat (liver, giblets, intestine) (one portion, 100g/ 2 soup ladles)

- □ never
- □ once a month or less frequent
- □ 2-3 times a month
- □ 1-2 times a week
- □ 3 times a week or more frequent

Meatballs, Hamburger patties, meatloaf (whole or one slice)

- □ never
- □ once a month or less frequent
- □ 2-3 times a month
- □ 1-2 times a week
- □ 3 times a week or more frequent

Fried sausage (e.g. khebab, Italian sausage, sausage meat) (1 piece)

- □ never
- □ once a month or less frequent
- □ 2-3 times a month
- □ 1-2 times a week
- □ 3 times a week or more frequent

Boiled sausage (e.g. Wiener, Frankfurters) (whole)

- □ never
- □ once a month or less frequent
- □ 2-3 times a month
- □ 1-2 times a week
- □ 3 times a week or more frequent

Dry and cured meat (e.g. bacon, kassler, prosciutto, bresaola) (one portion, 100g/ 2 soup ladles)

- □ never
- □ once a month or less frequent
- □ 2-3 times a month
- □ 1-2 times a week
- □ 3-4 times a week
- □ 5-6 times a week
- □ 1 time a day or more frequent

Dry sausage (e.g. Salami, chorizo) (one portion, 100g/ 2 soup ladles)

- □ never
- □ once a month or less frequent
- □ 2-3 times a month
- □ 1-2 times a week
- □ 3-4 times a week
- □ 5-6 times a week
- □ 1 time a day or more frequent

Corned beef (one portion, 100g/ 2 soup ladles)	◻ never<br>◻ once a month or less frequent<br>◻ 2-3 times a month<br>◻ 1-2 times a week	◻ 3-4 times a week<br>◻ 5-6 times a week<br>◻ 1 time a day or more frequent
Liverwurst, liver paté (one portion, 100g/ 2 soup ladles)	◻ never<br>◻ once a month or less frequent<br>◻ 2-3 times a month	◻ 1-2 times a week<br>◻ 3-4 times a week<br>◻ 5 times a week or more frequent

Fish

Fatty fish (e.g. sardines, anchovy, herring, tuna, mackerel, salmon, eel, trout) (fresh, canned, smoked) (one portion/ sardine-tin size)	◻ never<br>◻ once a month or less frequent<br>◻ 2-3 times a month	◻ 1-2 times a week<br>◻ 3 times a week or more frequent
Lean fish (e.g. cod, tilapia, catfish, pangasius, pollack, porgies, bass, barbell, pike) (one portion/ sardine-tin size)	◻ never<br>◻ once a month or less frequent<br>◻ 2-3 times a month	◻ 1-2 times a week<br>◻ 3 times a week or more frequent
Fish preparations (e. g. fish sticks, fishcake, preserved fish) (one portion/ sardine-tin size)	◻ never<br>◻ once a month or less frequent<br>◻ 2-3 times a month	◻ 1-2 times a week<br>◻ 3 times a week or more frequent

Shell fish (e.g. clams, mussels, oysters, scallops, shrimps, prawns, lobster, crayfish) (one portion/ sardine-tin size)	◻ never<br>◻ once a month or less frequent<br>◻ 2-3 times a month	◻ 1-2 times a week<br>◻ 3 times a week or more frequent

Mixed Dishes

Pizza (one portion/ one-eighth)	◻ never<br>◻ once a month or less frequent<br>◻ 2-3 times a month	◻ 1-2 times a week<br>◻ 3 times a week or more frequent
Tofu (soy protein, soy flour, meat-substitutes) (one portion/ sardine-tin size)	◻ never<br>◻ once a month or less frequent<br>◻ 2-3 times a month	◻ 1-2 times a week<br>◻ 3 times a week or more frequent

Cakes and Sweets

Please indicate your child’s average intake of the following food items during the past 6 months.

Cakes

Tart, Pie (e.g. with apple, plums etc.) (1 piece/ one-eighth)	□ never<br>□ once a month or less frequent<br>□ 2-3 times a month	□ 1-2 times a week<br>□ 3-4 times a week<br>□ 5 times a week or more frequent
Yeast cake, pastry, cake without filling (meat pie, rock buns, donut, cinnamon buns, coffee cake) (1 piece)	□ never<br>□ once a month or less frequent<br>□ 2-3 times a month	□ 1-2 times a week<br>□ 3-4 times a week<br>□ 5 times a week or more frequent
Sponge cake, cake with cream or custard filling, cream pie, cheesecake (1 piece/ 3 table spoons)	□ never<br>□ once a month or less frequent<br>□ 2-3 times a month	□ 1-2 times a week<br>□ 3-4 times a week<br>□ 5 times a week or more frequent
Whipped cream (1 tablespoon)	□ never<br>□ once a month or less frequent<br>□ 2-3 times a month	□ 1-2 times a week<br>□ 3-4 times a week<br>□ 5 times a week or more frequent

Sweets

Cookies, biscuits (e.g. britania, nuvita, manji, nutro) (3 pieces)	◻ never<br>◻ once a month or less frequent<br>◻ 2-3 times a month	◻ 1-2 times a week<br>◻ 3-4 times a week<br>◻ 5 times a week or more frequent
Chocolate and chocolate candy (50g, 2 pieces)	◻ never<br>◻ once a month or less frequent<br>◻ 2-3 times a month<br>◻ 1-2 times a week	◻ 3-4 times a week<br>◻ 5-6 times a week<br>◻ 1 time a day or more frequent
Candy, toffee (a handful)	◻ never<br>◻ once a month or less frequent<br>◻ 2-3 times a month<br>◻ 1-2 times a week	◻ 3-4 times a week<br>◻ 5-6 times a week<br>◻ 1 time a day or more frequent

Beverages

Please indicate your child's average intake of the following drinks during the past 6 monthsNote: Make sure that the respondent states the correct portion size. Please refer to the examples on the help sheet.

Water

(Sparkling) Bottled, tap or rain water
(1 glass = 300ml)

- ◻ never
- ◻ once a month or less frequent
- ◻ 2-3 times a month
- ◻ 1-3 times a week
- ◻ 4-6 times a week
- ◻ 1-2 times a day
- ◻ 3-4 times a day
- ◻ 5-6 times a day
- ◻ 7-8 times a day
- ◻ 9-10 times a day
- ◻ 11 times a day or more frequent

Sodas

Regular sodas, soft drinks, ice tea
(1 glass = 300ml)

- ◻ never
- ◻ once a month or less frequent
- ◻ 2-3 times a month
- ◻ 1-3 times a week
- ◻ 4-6 times a week
- ◻ 1-2 times a day
- ◻ 3-4 times a day
- ◻ 5-6 times a day
- ◻ 7-8 times a day
- ◻ 9-10 times a day
- ◻ 11 times a day or more frequent

Diet soda ('Light' soft-drinks e.g coke zero) (1 glass = 300ml)	□ never<br>□ once a month or less frequent<br>□ 2-3 times a month<br>□ 1-3 times a week<br>□ 4-6 times a week<br>□ 1-2 times a day	□ 3-4 times a day<br>□ 5-6 times a day<br>□ 7-8 times a day<br>□ 9-10 times a day<br>□ 11 times a day or more frequent
Non-alcoholic beer, light beer, ginger beer, root beer	□ never<br>□ once a month or less frequent<br>□ 2-3 times a month<br>□ once a week	□ 2-3 times a week<br>□ 4-6 times a week<br>□ once a day<br>□ twice a day or more often
	Portion size: □ ½ □ 1 □ 2 □ 3 bottle 0.5 L	

Juices

100 % Fruit juice (incl. home-made juices) (200ml)	□ never<br>□ once a month or less frequent<br>□ 2-3 times a month<br>□ 1-3 times a week<br>□ 4-6 times a week<br>□ 1-2 times a day	□ 3-4 times a day<br>□ 5-6 times a day<br>□ 7-8 times a day<br>□ 9-10 times a day<br>□ 11 times a day or more frequent

Fruit nectar and mixtures (e.g. afya) (200ml)	□ never	□ 3-4 times a day
	□ once a month or less frequent	□ 5-6 times a day
	□ 2-3 times a month	□ 7-8 times a day
	□ 1-3 times a week	□ 9-10 times a day
	□ 4-6 times a week	□ 11 times a day or more frequent
	□ 1-2 times a day	
Vegetable juice (e.g. carrot juice, tomato juice) (200ml)	□ never	□ 3-4 times a day
	□ once a month or less frequent	□ 5-6 times a day
	□ 2-3 times a month	□ 7-8 times a day
	□ 1-3 times a week	□ 9-10 times a day
	□ 4-6 times a week	□ 11 times a day or more frequent
	□ 1-2 times a day	

Coffee and Tea

Regular coffee (e.g. Nescafé) (1 cup)	□ never	□ 3-4 times a day
	□ once a month or less frequent	□ 5-6 times a day
	□ 2-3 times a month	□ 7-8 times a day
	□ 1-3 times a week	□ 9-10 times a day
	□ 4-6 times a week	□ 11 times a day or more frequent
	□ 1-2 times a day	

Decaffeinated coffee (1 cup)	◻ never<br>◻ once a month or less frequent<br>◻ 2-3 times a month<br>◻ 1-3 times a week<br>◻ 4-6 times a week<br>◻ 1-2 times a day	◻ 3-4 times a day<br>◻ 5-6 times a day<br>◻ 7-8 times a day<br>◻ 9-10 times a day<br>◻ 11 times a day or more frequent
Tea, black or green (e.g. Lipton) (1 cup)	◻ never<br>◻ once a month or less frequent<br>◻ 2-3 times a month<br>◻ 1-3 times a week<br>◻ 4-6 times a week<br>◻ 1-2 times a day	◻ 3-4 times a day<br>◻ 5-6 times a day<br>◻ 7-8 times a day<br>◻ 9-10 times a day<br>◻ 11 times a day or more frequent
Fruit-, Herbal tea (1 cup)	◻ never<br>◻ once a month or less frequent<br>◻ 2-3 times a month<br>◻ 1-3 times a week<br>◻ 4-6 times a week<br>◻ 1-2 times a day	◻ 3-4 times a day<br>◻ 5-6 times a day<br>◻ 7-8 times a day<br>◻ 9-10 times a day<br>◻ 11 times a day or more frequent

How did you usually drink your coffee or tea? Multiple answers are possible!

Coffee	◻ don't drink coffee	◻ plain	◻ with milk
	◻ with dairy creamer	◻ with sugar	◻ with sweetener
Tea, black, green	◻ don't drink tea	◻ plain	◻ with milk
	◻ with honey	◻ with sugar	◻ with sweetener
Fruit-, herbal tea	◻ don't drink tea	◻ plain	◻ with sweetener
	◻ with honey	◻ with sugar	

Fats

For this last section, please let me know what the usual fat content of the milk products your child consumed were and what your child's average intake of fats, oils and sauces for each milk product was during the past 6 months.

Fat content

Milk	◻ don't drink milk	Yoghurt	◻ don't eat yoghurt
	[◻ very low fat (0.1%)]		◻ fat free (0.1%)
	◻ skim (0.3%)		[◻ skim (0.3%)]
	◻ low fat (1.2%-1.5%)		◻ low fat (1.2%-1.5%)
	◻ whole/ direct from animal (3.5%)		◻ whole/ direct from animal (3.5%)
	◻ don't know/it varies		◻ don't know/it varies

Soft cheese	◻ don’t eat soft cheese	◻ regular	◻ low fat	◻ don’t know/it varies
Semi-soft and firm cheese	◻ don’t eat semi-soft and firm cheese	◻ regular	◻ low fat	◻ don’t know/it varies
Meat	◻ don’t eat meat	◻ regular	◻ low fat	◻ don’t know/it varies
Meat products	◻ don’t eat meat products	◻ regular	◻ low fat	◻ don’t know/it varies

Fats and Oils – How often did you prepare or eat meat and fish with any of the following fats?

Palm nut oil, palm kernel oil	◻ never	◻ rarely	◻ frequently	◻ always	◻ don’t know
Groundnut paste, peanut butter, shea butter	◻ never	◻ rarely	◻ frequently	◻ always	◻ don’t know
Butter	◻ never	◻ rarely	◻ frequently	◻ always	◻ don’t know
Margarine	◻ never	◻ rarely	◻ frequently	◻ always	◻ don’t know
Cooking fat (e.g. animal fats like lard or speck)	◻ never	◻ rarely	◻ frequently	◻ always	◻ don’t know
Olive oil	◻ never	◻ rarely	◻ frequently	◻ always	◻ don’t know
Other oils (e.g. vegetable, sunflower, canola)	◻ never	◻ rarely	◻ frequently	◻ always	◻ don’t know

How often did you eat or cook vegetables with any of the following fats?

Palm nut oil, palm kernel oil	◻ never	◻ rarely	◻ frequently	◻ always	◻ don’t know
Groundnut paste, peanut butter, shea butter	◻ never	◻ rarely	◻ frequently	◻ always	◻ don’t know
Butter	◻ never	◻ rarely	◻ frequently	◻ always	◻ don’t know
Margarine	◻ never	◻ rarely	◻ frequently	◻ always	◻ don’t know
Cooking fat (e.g. animal fats like lard or speck)	◻ never	◻ rarely	◻ frequently	◻ always	◻ don’t know
Olive oil	◻ never	◻ rarely	◻ frequently	◻ always	◻ don’t know
Other oils (e.g. vegetable, sunflower, canola)	◻ never	◻ rarely	◻ frequently	◻ always	◻ don’t know

Sauce – How often did you eat the following foods with sauce?

Sauce to meat or fish	◻ never	◻ rarely	◻ frequently	◻ always	◻ don’t know
Sauce to cooked vegetables	◻ never	◻ rarely	◻ frequently	◻ always	◻ don’t know
Sauce to pasta or rice	◻ never	◻ rarely	◻ frequently	◻ always	◻ don’t know

Salad Dressing – How often did you eat salad with any of the following ingredients?

Olive oil	◻ never	◻ rarely	◻ frequently	◻ always	◻ don’t know
Other oils (e.g. vegetable, linseed, sunflower, canola, safflower, germ oil)	◻ never	◻ rarely	◻ frequently	◻ always	◻ don’t know
Mayonnaise	◻ never	◻ rarely	◻ frequently	◻ always	◻ don’t know
Crème fraîche, cream, sour cream, remoulade	◻ never	◻ rarely	◻ frequently	◻ always	◻ don’t know
Yoghurt	◻ never	◻ rarely	◻ frequently	◻ always	◻ don’t know
Fresh herbs	◻ never	◻ rarely	◻ frequently	◻ always	◻ don’t know
Vinegar	◻ never	◻ rarely	◻ frequently	◻ always	◻ don’t know

End time ………………………………………………… (hh:mm) **(For interviewers only)**

Thank you for your time and your cooperation!
