# Supplementary material for: Reduced rank regression-derived dietary patterns related to climate-sensitive micronutrients and their associations with child undernutrition among young children in rural Kenya: findings from the ALIMUS study
Source: BMC Public Health. 2026 Jan 13;26:348. doi: 10.1186/s12889-026-26265-z (PMC12849572; doi:10.1186/s12889-026-26265-z)
Supplement: Supplementary file 2 — Supplementary Material 2 [file 12889_2026_26265_MOESM2_ESM.docx]

**Supplementary materials table S1. Characteristics of boys across quartiles of dietary pattern**

|  | | **Total**  **(n=339)** | **Quartiles of dietary pattern scores in boys** | | | | **P-value** |
| --- | --- | --- | --- | --- | --- | --- | --- |
|  |  |  | **Q1**  **(n=85)** | **Q2**  **(n=85)** | **Q3**  **(n=85)** | **Q4**  **(n=84)** |  |
| Mother age (year) | | 29 (24, 34) | 29.2 (24, 36) | 29 (25, 34) | 29 (22, 34) | 28 (24.5, 31.5) | 0.097 |
| Marital status | |  |  |  |  |  | 0.649 |
|  | Single | 84 (24.8) | 17 (20.2) | 24 (28.6) | 22 (26.2) | 21 (25.0) |  |
|  | Married | 255 (75.2) | 68 (26.7) | 61 (23.9) | 63 (24.7) | 63 (24.7) |  |
| Ethnic | |  |  |  |  |  | 0.474 |
|  | Luo | 315 (92.9) | 76 (24.1) | 81 (25.7) | 80 (25.4) | 78 (24.8) |  |
|  | Others | 24 (7.1) | 9 (37.5) | 4 (16.7) | 5 (20.8) | 6 (25.0) |  |
| Education level | |  |  |  |  |  | 0.024 |
|  | Less than primary | 221 (65.2) | 63 (28.5) | 58 (26.3) | 56 (25.3) | 44 (19.9) |  |
|  | Secondary or more | 118 (34.8) | 22 (18.6) | 27 (22.9) | 29 (24.6) | 40 (33.9) |  |
| Occupation | |  |  |  |  |  | 0.286 |
|  | Housewife | 67 (19.8) | 14 (20.9) | 20 (29.8) | 16 (23.9) | 17 (25.4) |  |
|  | Subsistence farmer | 183 (53.9) | 46 (25.1) | 46 (25.1) | 52 (28.4) | 39 (21.3) |  |
|  | Skilled workers/laborers | 67 (19.8) | 22 (32.8) | 14 (20.9) | 10 (14.9) | 21 (31.3) |  |
|  | Business | 22 (6.5) | 3 (13.6) | 5 (22.7) | 7 (31.8) | 7 (31.8) |  |
| Household size | | 6 (5, 7) | 6 (5, 7) | 6 (5, 7.5) | 6 (5, 7) | 5 (5, 7) | 0.056 |
| Number of under-fives in household | | 1 (1, 2) | 2 (1, 2) | 1.6 (1, 2) | 1 (1, 2) | 1 (1, 2) | 0.029 |
| Children's age (month) | | 15 (11, 19) | 11 (8, 16) | 15 (11, 20) | 16 (11, 19) | 17 (14, 20) | <0.001 |
| Protein-energy status | |  |  |  |  |  |  |
|  | Weight-for-age (WAZ) z-score | -0.47 (-1.31, 0.29) | -0.1 (-1.01, 0.33) | -0.38 (-1.22, 0.49) | -0.46 (-1.18, 0.23) | -0.75 (-1.44, -0.02) | 0.016 |
|  | Weight-for-height (WHZ) z-score | -0.02 (-0.81, 0.71) | 0.11 (-0.59, 0.83) | 0.05 (-0.86, 0.97) | -0.1 (-0.72, 0.51) | -0.37 (-1.07, 0.6) | 0.026 |
|  | Height-for-age (HAZ) z-score | -0.88 (-1.8, 0.03) | -0.59 (-1.51, 0.17) | -0.91 (-1.57, 0.06) | -0.69 (-1.65, 0.03) | -1.1 (-1.94, -0.1) | 0.131 |
|  | Underweight (WAZ ≤ -2) | 37 (10.9) | 8 (21.6) | 10 (27.0) | 10 (27.0) | 9 (24.4) | 0.955 |
|  | Wasting (WHZ ≤ -2) | 22 (6.5) | 6 (27.2) | 8 (36.4) | 4 (18.2) | 4 (18.2) | 0.552 |
|  | Stunting (HAZ ≤ -2) | 74 (21.8) | 15 (20.3) | 18 (24.3) | 21 (28.4) | 20 (27.0) | 0.683 |
| Macro- and micro-nutrient intake | |  |  |  |  |  |  |
|  | Total protein intake (g/day) | 32.1 (24.0, 39.2) | 18.5 (13.3, 21.2) | 27.9 (25.3, 33.1) | 33.9 (31.1, 36.9) | 42.4 (39.1, 48.1) | <0.001 |
|  | Total fat intake (g/day) | 28.7 (19.8, 38.3) | 14.4 (9.7, 19.7) | 23.2 (20.2, 30.7) | 31.2 (25.3, 34.8) | 42.6 (35.2, 48.3) | <0.001 |
|  | Total carbohydrate intake (g/day) | 158.2 (108.8, 201.7) | 81.8 (58.2, 102.5) | 140.2 (116.4, 172.6) | 172.9 (137.2, 200.7) | 222.0 (192.1, 259.1) | <0.001 |
|  | Total energy intake (kcal/day) | 1047.5 (742.2, 1329.6) | 547.4 (400.3, 659.8) | 892.0 (772.3, 1087.9) | 1108 (951.5, 1241.4) | 1473.9 (1297.9, 1622.8) | <0.001 |
|  | Fiber intake (g/day) | 14.9 (10.6, 18.1) | 8.6 (6.3, 11.2) | 13.5 (11.2, 16.4) | 15.7 (13.9, 17.1) | 18.9 (17.4, 22.1) | <0.001 |
|  | Iron intake (mg/day) | 7.4 (5.4, 8.7) | 4.2 (3.1, 5.2) | 6.6 (5.9, 7.5) | 8.1 (7.3, 8.6) | 9.6 (8.7, 10.6) | <0.001 |
|  | Zinc intake (mg/day) | 5.9 (4.2, 7.5) | 3.5 (2.7, 4.4) | 5.4 (4.5, 6.6) | 6.2 (5.5, 7.2) | 8.2 (7.1, 9.3) | <0.001 |
|  | Selenium intake (mg/day) | 48.1 (31.4, 65.3) | 24.9 (18.0, 34.0) | 42.9 (31.7, 55.5) | 52.6 (40.5, 62.1) | 66.8 (57.6, 79.7) | <0.001 |
|  | Vitamin A intake (µ/day) | 76.2 (45.4, 144.5) | 39.6 (21.6, 66.1) | 68.5 (45.5, 134.7) | 93.4 (52.2, 140.7) | 143.4 (77.9, 176.2) | <0.001 |
|  | ß-carotene intake (µ/day) | 3990.2 (2603.1, 5336.7) | 1827.3 (1269.4, 2468.3) | 3502.3 (2841.2, 4290.3) | 4387.7 (3847.0, 6008.0) | 5957.7 (4827.8, 7466.4) | <0.001 |
|  | Retinol equivalent (µ/day) | 448.5 (285.2, 598.0) | 219.4 (148.5, 276.4) | 394.3 (316.7, 473.1) | 492.3 (426.5, 601.6) | 649.0 (552.6, 827.3) | <0.001 |
| Data are expressed as median (interquartile range) for continuous variables and number (percentage) for categorical variables.  P-values were analyzed using nonparametric test for trend for continuous variables and Chi-Square for categorical variables | | | | | | | |

**Supplementary materials table S2. Characteristics of girls across quartiles of dietary pattern**

|  | | **Total**  **(n=287)** | **Quartiles of dietary pattern scores in girls** | | | | **P-value** |
| --- | --- | --- | --- | --- | --- | --- | --- |
|  |  |  | **Q1**  **(n=72)** | **Q2**  **(n=72)** | **Q3**  **(n=72)** | **Q4**  **(n=72)** |  |
| Mother age (year) | | 29.2 (25, 34) | 31 (27, 34.5) | 28 (21.5, 33) | 29 (24.5, 34) | 31 (25, 35) | 0.876 |
| Marital status | |  |  |  |  |  | 0.014 |
|  | Single | 58 (20.2) | 10 (17.2) | 19 (32.8) | 21 (36.2) | 8 (13.8) |  |
|  | Married | 229 (79.8) | 62 (27.1) | 53 (23.1) | 51 (22.3) | 63 (27.5) |  |
| Ethnic | |  |  |  |  |  | 0.049 |
|  | Luo | 271 (94.4) | 67 (24.7) | 64 (23.6) | 71 (26.2) | 69 (25.5) |  |
|  | Others | 16 (5.6) | 5 (31.3) | 8 (50.0) | 1 (6.2) | 2 (12.5) |  |
| Education level | |  |  |  |  |  | 0.004 |
|  | Less than primary | 193 (67.2) | 59 (30.6) | 49 (25.4) | 47 (24.3) | 38 (19.7) |  |
|  | Secondary or more | 94 (32.8) | 13 (13.8) | 23 (24.5) | 25 (26.6) | 33 (35.1) |  |
| Occupation | |  |  |  |  |  | 0.239 |
|  | Housewife | 51 (17.8) | 12 (23.5) | 16 (31.4) | 14 (27.5) | 9 (17.6) |  |
|  | Subsistence farmer | 156 (54.3) | 48 (30.8) | 32 (20.5) | 35 (22.4) | 41 (26.3) |  |
|  | Skilled workers/laborers | 58 (20.2) | 9 (15.5) | 19 (32.8) | 16 (27.6) | 14 (24.1) |  |
|  | Business | 22 (7.7) | 3 (13.7) | 5 (22.7) | 7 (31.8) | 7 (31.8) |  |
| Household size | | 6 (5, 7) | 6 (5, 7) | 6 (5, 7) | 6 (5, 7) | 6 (5, 7) | 0.902 |
| Number of under-fives in household | | 2 (1, 2) | 2 (1, 2) | 2 (1, 2) | 2 (1, 2) | 2 (1, 2) | 0.647 |
| Children age (month) | | 15 (10, 19) | 9.5 (7, 17) | 14.5 (11, 19.5) | 16 (11, 20) | 17 (14, 20) | <0.001 |
| Protein-energy status | |  |  |  |  |  |  |
|  | Weight-for-age (WAZ) z-score | -0.2 (-0.91, 0.6) | -0.23 (-1.17, 0.53) | -0.13 (-0.83, 0.93) | -0.13 (-0.64, 0.55) | -0.33 (-1.06, 0.41) | 0.753 |
|  | Weight-for-height (WHZ) z-score | 0.18 (-0.62, 0.8) | 0.1 (-0.8, 0.69) | 0.1 (-0.60, 0.97) | 0.37 (-0.44, 0.93) | -0.03 (-0.62, 0.79) | 0.443 |
|  | Height-for-age (HAZ) z-score | -0.54 (-1.47, 0.44) | -0.66 (-1.44, 0.44) | -0.4 (-1.52, 0.66) | -0.5 (-1.69, 0.41) | -0.6 (-1.26, -0.04) | 0.549 |
|  | Underweight (WAZ ≤ -2) | 14 (4.9) | 5 (35.7) | 3 (21.4) | 3 (21.4) | 3 (21.4) | 0.829 |
|  | Wasting (WHZ ≤ -2) | 8 (2.8) | 3 (37.5) | 2 (25.0) | 2 (25.0) | 1 (12.5) | 0.800 |
|  | Stunting (HAZ ≤ -2) | 38 (13.2) | 7 (18.4) | 13 (34.2) | 13 (34.2) | 5 (13.2) | 0.109 |
| Macro- and micro-nutrient intake | |  |  |  |  |  |  |
|  | Total protein intake (g/day) | 31.1 (21.3, 38.0) | 16.1 (12.1, 21.3) | 26.5 (23.3, 32.3) | 34.4 (29.9, 37.4) | 42.2 (39.3, 46.9) | <0.001 |
|  | Total fat intake (g/day) | 26.5 (17.5, 36.7) | 13.4 (8.7, 17.2) | 24.6 (18.3, 31.3) | 27.2 (23.8, 34.0) | 39.6 (34.5, 46.2) | <0.001 |
|  | Total carbohydrate intake (g/day) | 151.7 (100.0, 212.4) | 73.3 (57.3, 107.1) | 134.6 (105.1, 154.1) | 169.7 (148.9, 191.9) | 249.9 (219.0, 275.4) | <0.001 |
|  | Total energy intake (kcal/day) | 991.2 (662.2, 1364.4) | 489.5 (366.7, 647.6) | 884.3 (722.3, 1008.6) | 1057.2 (948.1, 1258.5) | 1561.2 (1428.9, 1681.6) | <0.001 |
|  | Fiber intake (g/day) | 14.4 (9.8, 18.3) | 7.5 (6.0, 10.4) | 12.6 (10.6, 15.8) | 15.6 (13.1, 17.8) | 20.0 (16.9, 23.3) | <0.001 |
|  | Iron intake (mg/day) | 7.0 (5.0, 8.7) | 3.9 (3.0, 5.1) | 6.2 (5.3, 7.0) | 8.2 (7.1, 8.7) | 9.6 (8.6, 10.8) | <0.001 |
|  | Zinc intake (mg/day) | 6.0 (4.0, 7.4) | 3.1 (2.4, 4.1) | 5.3 (4.4, 6.3) | 6.4 (5.4, 7.1) | 8.4 (7.4, 9.5) | <0.001 |
|  | Selenium intake (mg/day) | 50.1 (32.5, 68.5) | 25.6 (17.2, 40.2) | 42.4 (33.3, 70.5) | 53.8 (42.8, 64.1) | 66.2 (57.5, 79.3) | <0.001 |
|  | Vitamin A intake (µ/day) | 76.8 (43.7, 130.2) | 34.7 (23.0, 55.4) | 76.2 (52.2, 141.4) | 79.2 (51.8, 138.9) | 114.2 (78.6, 158.8) | <0.001 |
|  | ß-carotene intake (µ/day) | 3588.8 (2483.2, 5510.1) | 1949.1 (1177.7, 2614.2) | 3239.3 (2563.7, 3622.1) | 4250.6 (3469.6, 5403.0) | 7210.2 (5364.5, 8943.6) | <0.001 |
|  | Retinol equivalent (µ/day) | 424.4 (273.5, 585.1) | 212.5 (142.6, 276.4) | 350.3 (288.4, 450.4) | 480.3 (402.5, 572.4) | 741.9 (570.4, 864.0) | <0.001 |
| Data are expressed as median (interquartile range) for continuous variables and number (percentage) for categorical variables.  P-values were analyzed using nonparametric test for trend for continuous variables and Chi-Square for categorical variables | | | | | | | |

**Supplementary materials table S3. Sensitivity analysis of associations between dietary pattern scores and anthropometric z-scores among girls and boys**

|  | **Model 3** | | **Model 3+** | |
| --- | --- | --- | --- | --- |
|  | **ß (95% CI)** | **p-value** | **ß (95% CI)** | **p-value** |
| Girls | | | | |
| **Weight-for-age (WAZ) z-score** |  |  |  |  |
| Per 1 score-point increase | 0.19 (0.02, 0.36) | 0.033 | 0.20 (0.03, 0.38) | 0.024 |
| Quartile 1 | Ref | | | |
| Quartile 2 | 0.68 (0.24, 1.12) | 0.003 | 0.68 (0.24, 1.13) | 0.003 |
| Quartile 3 | 0.69 (0.16, 1.21) | 0.010 | 0.70 (0.17, 1.22) | 0.010 |
| Quartile 4 | 0.86 (0.13, 1.58) | 0.021 | 0.88 (0.15, 1.61) | 0.019 |
| *p-value for trend* |  | 0.022 |  | 0.021 |
| **Weight-for-height (WHZ) z-score** |  |  |  |  |
| Per 1 score-point increase | 0.16 (-0.01, 0.34) | 0.071 | 0.18 (-0.00, 0.35) | 0.054 |
| Quartile 1 | Ref | | | |
| Quartile 2 | 0.54 (0.09, 0.98) | 0.019 | 0.55 (0.10, 0.99) | 0.017 |
| Quartile 3 | 0.76 (0.23, 1.29) | 0.005 | 0.78 (0.25, 1.32) | 0.004 |
| Quartile 4 | 0.83 (0.10, 1.57) | 0.027 | 0.87 (0.13, 1.61) | 0.022 |
| *p-value for trend* |  | 0.040 |  | 0.034 |
| **Height-for-age (HAZ) z-score** |  |  |  |  |
| Per 1 score-point increase | 0.14 (-0.06, 0.35) | 0.167 | 0.15 (-0.05, 0.36) | 0.147 |
| Quartile 1 | Ref | | | |
| Quartile 2 | 0.53 (0.01, 1.05) | 0.047 | 0.52 (-0.00, 1.04) | 0.051 |
| Quartile 3 | 0.22 (-0.40, 0.83) | 0.490 | 0.20 (-0.42, 0.82) | 0.520 |
| Quartile 4 | 0.44 (-0.41, 1.30) | 0.312 | 0.44 (-0.42, 1.30) | 0.318 |
| *p-value for trend* |  | 0.196 |  | 0.201 |
| Boys | | | | |
| **Weight-for-age (WAZ) z-score** |  |  |  |  |
| Per 1 score-point increase | -0.06 (-0.23, 0.11) | 0.508 | -0.06 (-0.23, 0.11) | 0.488 |
| Quartile 1 | Ref | | | |
| Quartile 2 | 0.09 (-0.39, 0.56) | 0.718 | -0.00 (-0.48, 0.48) | 0.988 |
| Quartile 3 | -0.15 (-0.68, 0.37) | 0.575 | -0.23 (-0.76, 0.31) | 0.403 |
| Quartile 4 | -0.16 (-0.85, 0.53) | 0.648 | -0.22 (-0.91, 0.47) | 0.527 |
| *p-value for trend* |  | 0.674 |  | 0.712 |
| **Weight-for-height (WHZ) z-score** |  |  |  |  |
| Per 1 score-point increase | -0.10 (-0.27, 0.07) | 0.262 | -0.10 (-0.27, 0.07) | 0.244 |
| Quartile 1 | Ref | | | |
| Quartile 2 | -0.10 (-0.57, 0.36) | 0.671 | -0.20 (-0.67, 0.27) | 0.410 |
| Quartile 3 | -0.25 (-0.77, 0.27) | 0.340 | -0.33 (-0.85, 0.19) | 0.211 |
| Quartile 4 | -0.30 (-0.98, 0.37) | 0.377 | -0.37 (-1.04, 0.30) | 0.281 |
| *p-value for trend* |  | 0.765 |  | 0.655 |
| **Height-for-age (HAZ) z-score** |  |  |  |  |
| Per 1 score-point increase | 0.02 (-0.19, 0.22) | 0.885 | 0.02 (-0.19, 0.22) | 0.887 |
| Quartile 1 | Ref | | | |
| Quartile 2 | 0.28 (-0.28, 0.83) | 0.325 | 0.23 (-0.33, 0.79) | 0.422 |
| Quartile 3 | 0.05 (-0.56, 0.67) | 0.868 | 0.01 (-0.61, 0.64) | 0.967 |
| Quartile 4 | 0.03 (-0.77, 0.84) | 0.934 | 0.00 (-0.80, 0.81) | 0.994 |
| *p-value for trend* |  | 0.631 |  | 0.721 |
| Model 1: adjusted for age  Model 2: model 1 + mother's age, education, occupation, marital status, number of children under five, and household size  Model 3: model 2 + total energy intake (kcal/day), fibre intake (g/d), and breastfeeding status  Model 3+: adjusted for model 3 + past 4 weeks of fever and diarrhea | | | | |
